# Two Complete Mitochondrial Genomes of *Leptobrachium* (Anura: Megophryidae: Leptobrachiinae): Characteristics, Population Divergences, and Phylogenetic Implications

**DOI:** 10.3390/genes14030768

**Published:** 2023-03-21

**Authors:** Qiang Zhou, Hong-Mei Xiang, Ming-Yao Zhang, Ying Liu, Zhi-Rong Gu, Xiang-Ying Lan, Jin-Xiu Wang, Wan-Sheng Jiang

**Affiliations:** 1 Hunan Engineering Laboratory for Chinese Giant Salamander’s Resource Protection and Comprehensive Utilization, Key Laboratory of Hunan Forest Products and Chemical Industry Engineering, National and Local United Engineering Laboratory of Integrative Utilization Technology of Eucommia ulmoides, Jishou University, Zhangjiajie 427000, China; zq1559534582@126.com (Q.Z.); hmxiang@163.com (H.-M.X.);; 2 College of Biology and Environmental Sciences, Jishou University, Jishou 416000, China; 3 Zhangjiajie National Forest Park, Zhangjiajie 427400, China; 4 National Nature Reserve of Badagongshan, Zhangjiajie 427100, China

**Keywords:** Megophryidae, mitochondrial genome, next-generation sequencing, phylogenetic analysis

## Abstract

The mustache toads *Leptobrachium boringii* and *Leptobrachium liui* are two attractive species in Megophryidae, in which adult males have mustache-like keratinized nuptial spines on their upper lip. However, both are under threat due to multiple factors, of which scientific studies are still very limited. In this study, two new complete mitochondrial genomes of *L. boringii* and *L. liui* were sequenced, assembled, and annotated based on next-generation sequencing. The mitogenome lengths of *L. boringii* and *L. liui* were found to be 17,100 and 17,501 bp, respectively, with both containing 13 protein coding genes, 23 tRNAs, 2 rRNAs, and 1 non-coding control region. Nucleotide diversity analyses indicate that *atp8*, *atp6*, and *nad2* showed higher nucleotide diversity than *cox1*, *cox3,* and *cytb*. The intraspecific genetic distances among three different populations of *L. boringii* exceed 4%, and those between two populations of *L. liui* reach 7%. Phylogenetic relationships support their division into two subfamilies of Megophryidae (Leptobrachiinae and Megophryinae) as well as two species groups within *Leptobrachium,* corresponding to the number of keratinized nuptial spines (10–48 in the *L. boringii* species group vs. 2–6 in the *L. liui* species group). The two new mitogenomes reported in this study provide valuable data for future molecular evolutionary and conservation studies of the genus *Leptobrachium* and other Megophryidae toads.

## 1. Introduction

The toads of the family Megophryidae (Bonaparte, 1850) represent a group endemic to Asia and are found mainly distributed in tropical and subtropical forestry regions, ranging from northeast India, east Himalayas, eastward to South China, and southward to Southeast Asia [1]. For a long time, Megophryidae was known as Megophryinae, a subfamily within Pelobatidae (Bonaparte, 1850), until morphological studies showed that Megophryinae should be excluded from Pelobatidae based on skeletal structures; thus, it was suggested it be promoted to family level [2,3]. The validity and monophyly of Megophryidae has been supported by subsequent molecular phylogenetic studies [4,5]. At present, Megophryidae is widely recognized as an independent family that includes two subfamilies, the Leptobrachiinae and the Megophryinae, which comprise 179 and 129 species, respectively [6]. 

According to AmphibiaWeb (2023), *Leptobrachium* (Tschudi, 1838), the type genus of the subfamily Leptobrachiinae, includes 38 species of which 11 have been recorded in China, with 9 being endemic [7]. The toads in *Leptobrachium* prefer montane habitats in broad-leaf temperate forests at elevations of 700~1700 m, and they usually require slow-flowing streams with dense vegetation for breeding and forested terrestrial uplands to occupy outside the breeding season [7,8,9]. The taxonomy within the genus *Leptobrachium* is, however, debatable to some extent. It was originally divided into two subgenera, *Vibrissaphora* and *Leptobrachium*, according to whether or not adult males have keratinized nuptial spines [10,11]. There were also studies that further treated the spines in adult males as a basis for distinguishing *Vibrissaphora* and *Leptobrachium* as two distinct genera [7,12]. However, recent phylogenetic studies have proposed a single genus, *Leptobrachium,* including all species without subgeneric division, for the reason that *Vibrissaphora* species were not recovered as a single monophylum but dispersed within the lineage of *Leptobrachium* [8,13,14]. *Leptobrachium* species have a pair of large dark eyes with the upper half of the iris extraordinarily colored, such as sky-blue or green. Since these toads always hide in the daytime and come out at night, a study on their retina showed that the cell organization is principally adapted to enable sight at low light intensities [15]. This characteristic also seems to be useful for species identification and may bear phylogenetic signals; however, more data support for this speculation is still required [14]. Another unique aspect of the genus is its choice of cold season for breeding, whereas most frogs and toads breed in warmer months [16]. 

**Figure 1:**
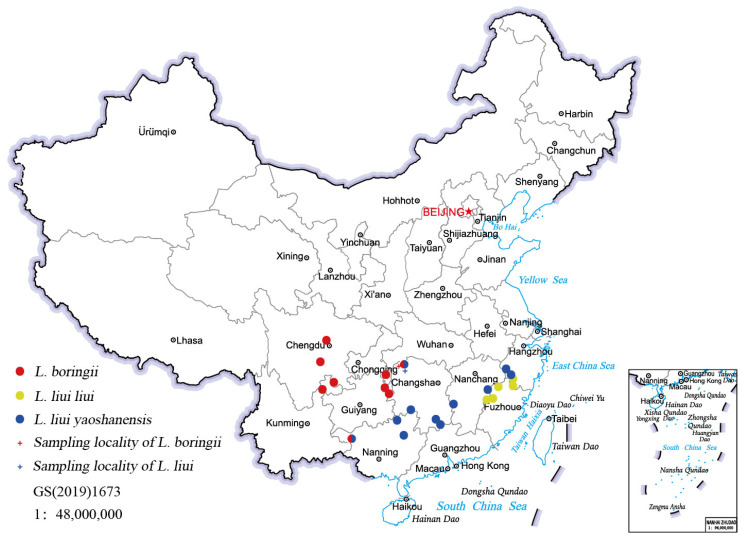
The recorded distribution sites of *L. boringii* and *L. liui* (data from [7]).

*Leptobrachium boringii* (Liu, 1945), known as the Emei mustache toad, is an attractive species in the genus. It was named after the mustache the males use as weapon for fighting during the breeding seasons, which is actually small keratinized nuptial spines growing on the upper lip [17]. The males are aggressive toward each other and outgrow the females to also aid in male–male combat, which is pretty rare in anurans [18]. Studies on reproductive behavior have indicated that males provide paternal care and larger males have higher mating success [19,20]. Both male combat and paternal care have been suggested to have adaptive significance in competing for nests, improving mating opportunities, and offspring survival [18,19,21]. Adult males of another species, *L. liui* (Pope, 1947), also have keratinized spines (mustache) on the upper lip but less in number than *L. boringii* (2–4 vs. 10–16) [22]. The distribution ranges of *L. liui* and *L. boringii* are basically separate but overlap at a few boundary areas (Figure 1). *Leptobrachium liui* is known as having two subspecies, *L. liui liui* and *L. liui yaoshanensis*, with two and four keratinized spines, respectively [22]. 

Although most species in the *Leptobrachium* genus are only found very narrowly distributed in a few regions, *L. boringii* and *L. liui* are two exceptions that are found over a relatively large area. The current known populations of *L. boringii* are in Chongqing, Guangxi, Guizhou, Hunan, Yunnan, and Sichuan Provinces, and populations of *L. liui* are recorded in the Fujian, Zhejiang, Jiangxi, Hunan, Guangxi, and Guangdong Provinces of China (Figure 1; [7]). Although having relatively wide distribution, many populations of the two species are undergoing rapid decline due to over-catching, habitat loss, and degradation, among other factors [7,9]. The tadpoles of both *L. boringii* and *L. liui* take about four months to hatch and usually require three years to complete metamorphosis, which aggravates their chances of survival [22,23]. At present, *L. boringii* is classified as “Endangered” in the IUCN Red List of Threatened Species [24] and listed in the second class of National Key Protected Wild Animals of China [25], while *L. liui* was classified as “Least Concern” on the IUCN Red List of Threatened Species [26]. 

Speciation of amphibians has been driven by environmental factors and physiological adaptations during their short-term life cycle and long-term evolutionary history, which have shaped their current distribution and diversity patterns, showing similar characteristics or unique traits in morphology [22]. With more than 300 species in Megophryidae, a robust phylogenetic relationship reconstructed using mitogenomes would provide a basic framework to understand the phylogeny and trait evolution of this group. However, the available mitogenome data are still very limited. For instance, only 18 complete and 9 nearly complete mitogenomes of Megophryidae are available from NCBI to date. As for *L. boringii*, although it is relatively widely distributed, details of its population diversity and genetic differentiation are still largely unknown. There are two previous studies reporting the mitogenomes of *L. boringii* from two isolated populations: the Emei Mountains (EM) in Sichuan Province and Pengshui County (PS) in Chongqing Province [27,28]; however, these two mitogenomes were obtained using a traditional PCR-targeted sequencing method, and the sequences were incomplete. When it comes to *L. liui*, there was also a mitogenome reported from the population of the Jiulongshan National Nature Reserve (JLS) in Zhejiang Province. Similarly to *L. boringii*, however, it was only briefly described in the paper [29]. In this study, using next-generation sequencing (NGS) technology, we report two newly obtained complete mitogenomes of the genus *Leptobrachium*, one from *L. boringii* based on its easternmost population from the National Nature Reserve of Badagongshan in Sangzhi County (SZ) and the other from *L. liui* based on its northernmost population from the Zhangjiajie National Forest Park in Wulingyuan District (WLY). Both Sangzhi County and Wulingyuan District belong to Zhangjiajie City in Hunan Province and, interestingly, represent a boundary area of *L. boringii* and *L. liu* here (Figure 1). This study aims to: (1) analyze and describe the characteristics of the updated complete mitogenomes of *L. boringii* and *L. liui* in detail, (2) compare the genetic distances among the three representative populations of *L. boringii* and the two populations of *L. liui*, and (3) reconstruct the phylogenetic relationships of Megophryidae and present implications of this evolutionarily interesting group.

## 2. Materials and Methods

### 2.1. Sample Collection and Sequencing

The sample of *L. boringii* was collected in May 2021 from National Nature Reserve of Badagongshan, Sangzhi County, and the sample of *L. liui* was collected in September 2021 from Zhangjiajie National Forest Park, Wulingyuan District, both in Zhangjiajie City in Hunan Province, China. Permissions of the field survey for scientific purposes were approved by the local administration, and the collection of toads used in this study complied with the Wildlife Protection Law of China. According to the “3R principle” (Reduction, Replacement, and Refinement) of animal sampling, only one sample of each species was used in this study. All procedures of animal collection and treatment comply with the guidance of the Code of Practice for the Housing and Care of Animals. The specimens were euthanized and preserved in 95% alcohol as reference specimens, with a small liver sample used for molecular analysis. DNA extraction was conducted using the DNeasy Blood and Tissue Kit (Qiagen, Hilden, Germany), and a DNA library was then constructed using the VAHTS Universal DNA Library Prep Kit for Illumina V3 (Vazyme, Nanjing, China). High-throughput sequencing was performed in paired-end mode on the DNBSEQ-T7 platform (Complete Genomics and MGI Tech, Shenzhen, China), generating approximately 30 Gb of raw reads of 150 bp read length. 

### 2.2. Sequence Assembly, Annotation, and Analysis

The complete mitogenomes of *L. boringii* and *L. liui* were assembled by NOVOPlasty 4.3 [30] based on the raw reads produced through NGS, both using the *cytb* gene (1141 bp) as seed sequences, from the previous reported mitogenome of *L. boringii* (KJ630505). Then, the positions and directions of protein coding genes (PCGs), ribosomal RNA genes (rRNAs), transfer RNA genes (tRNAs), and the control region (D-loop) were annotated using MITOS Web Server [31]. The sites and secondary structure of tRNAs were further inferred with the help of ARWEN and Scan-SE [32]. Visualization and circularization of the complete mitogenome were performed through the online server GeSeq [33]. Other analyses, such as nucleotide composition, AT and GC skew, and the determination of sequence genetic distances under Kimura’s two-parameter (K2P) model [34], were conducted through MEGA X [35]. Relative synonymous codon usage (RSCU), nucleotide diversity (Pi), and the ratio of the nonsynonymous substitution rate (Ka) and the synonymous substitution rate (Ks) were all calculated through DnaSP 6 [36]. 

### 2.3. Phylogenetic Analysis

The sequences used for phylogenetic reconstruction consisted of *L. boringii* (SZ population) and *L. liui* (WLY population) sequences we obtained in this study and another 27 mitogenomes that were downloaded from NCBI, including 2 mitogenomes of *L. boringii* (EM and PS populations), 1 mitogenome of *L. liui* (JLS population), and 24 mitogenomes of other 21 representative species within Megophryidae. *Microhyla fissipes* in the Microhylidae family was used as the outgroup. Each of the 13 PCGs was extracted from the dataset of 30 mitogenomes and manually checked, and all PCGs were then aligned using the inbuilt MUSCLE module in MEGA X and concatenated to make a combined PCG dataset. To uncover the population status and phylogenetic position of *L. boringii* and *L. liui*, the phylogenetic analyses were reconstructed using both the maximum likelihood (ML) method conducted in RAxML 8.0.2 [37] and the Bayesian inference (BI) method via MrBayes 3.2.7 [38]. Partitioning scheme and nucleotide substitution models for ML and BI phylogenetic analyses were selected using PartitionFinder 2 [39] based on the Akaike information criterion (AIC). The statistical confidence was assessed through a bootstrap test with 1000 replicates in ML trees and posterior probability calculation of the BI trees that under simultaneously run for 1.0 × 10^7^ million generations, with sampling conducted every 1000 generations and discarding the initial 25% generations. 

## 3. Results

### 3.1. Mitogenome Annotation and Nucleotide Composition

**Figure 2:**
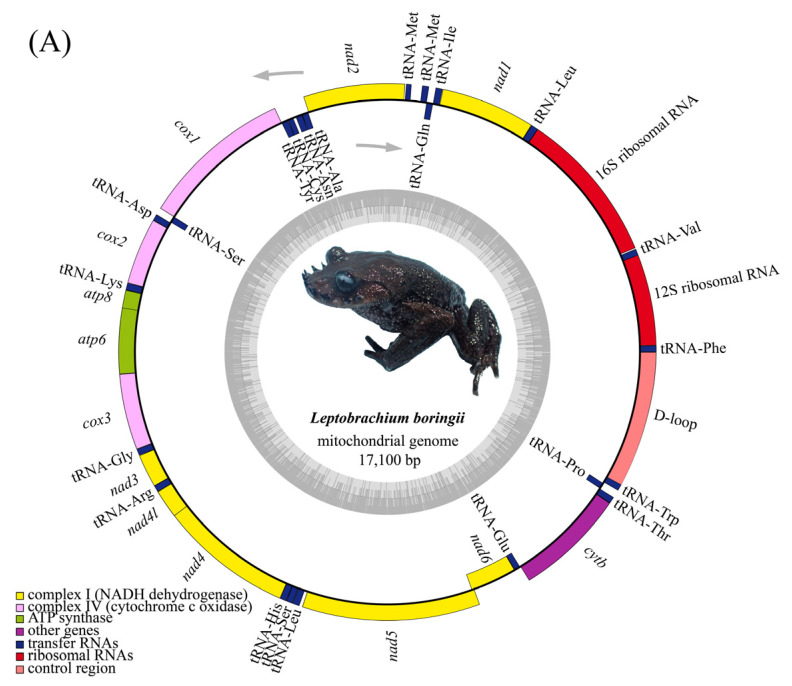

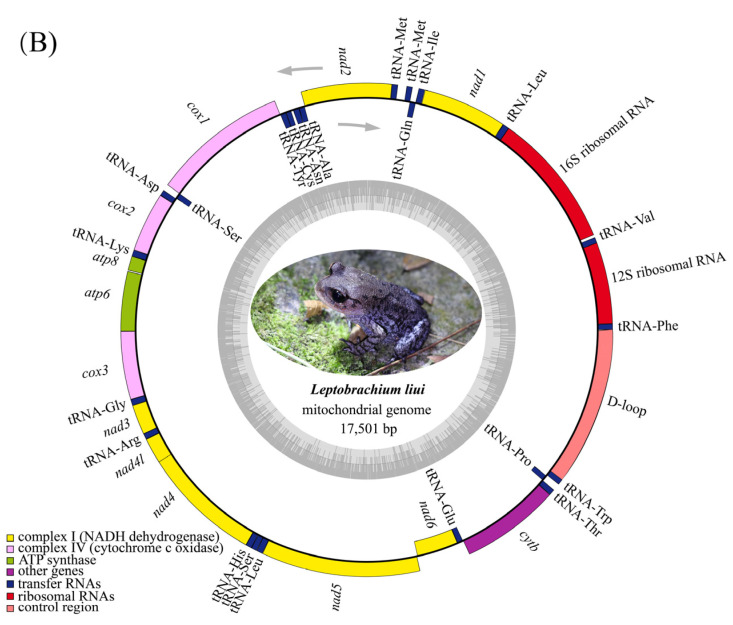
Gene map of the mitogenome of *L. boringii* (**A**) and *L. liui* (**B**).

The assembled complete mitogenomes of *L. boringii* and *L. liui* were 17,100 and 17,501 bp in length, which were deposited in NCBI under accession numbers OP373724 and OP503540, respectively. The mitogenomes of both species had a typical gene organization, with 13 PCGs, 2 rRNAs, 23 tRNAs, and a D-loop region (Table 1; Figure 2). There was a total of 187 and 174 bp of intergenic nucleotides (IGNs) dispersed in 12 and 13 locations of two species, ranging from 1 to 108 bp and 1 to 99 bp in length, respectively (Table 1). Similar to other species in Megophryidae, the overall base composition of *L. boringii* was 31.5% T, 25.5% C, 15.3% G, and 27.7% A, while that of *L. liui* was 32.7% T, 24.3% C, 14.8% G, and 28.1% A (Table 2), indicating an A+T bias with greater A+T than G+C content (59.2% vs. 40.8% and 60.8% vs. 39.1%). Additionally, both the AT and GC skew were negative for the mitogenomes of all Megophryidae species, reflecting a general bias toward T and C base pairs (Table 2). 

### 3.2. Characteristics of rRNAs, tRNAs, and the Control Region

In both *L. boringii* and *L. liui*, there were 23 tRNAs interspersed in the whole mitogenome, ranging from 64 to 75 bp, with tRNA^Cys^ being the shortest and tRNA^Leu^ the longest. Similarly to other congeners in the genus *Leptobrachium*, there was also a tandem duplication of tRNA^Met^ that was separated by long IGNs (intergenic nucleotides, 108 bp in *L. boringii* and 99 bp in *L. liui*). There were two rRNAs with a total length of 2520 bp in *L. boringii* and 2500 bp in *L. liui*. The 16S rRNA was located between tRNA^Val^ and tRNA^Leu^ with a length of 1580 bp in *L. boringii* and 1563 bp in *L. liui*, whereas the 12S rRNA was located between tRNA^Phe^ and tRNA^Val^ with 940 bp in *L. boringii* and 937 bp in *L. liui*. The non-coding domain of D-loop was located between tRNA^Trp^ and tRNA^Phe^, with a length of 1406 bp in *L. boringii* and 1815 bp in *L. liui* (Table 1). For both *L. boringii* and *L. liui*, most genes were encoded on the heavy (H) strand, except for the *nad6* and eight tRNA genes (tRNA^Gln^, tRNA^Ala^, tRNA^Asn^, tRNA^Cys^, tRNA^Tyr^, tRNA^Ser^, tRNA^Glu^, and tRNA^Pro^) encoded on the light (L) strand. 

Among all the 13 PCGs, the shortest was the *atp8* gene, being only 186 bp in *L. boringii* and 165 bp in *L. liui*, while the longest was *nad5* at 1833 bp in *L. boringii* and 1830 bp in *L. liui*. Three PCGs (*cox1*, *atp8*, *nad3*) started with GTG, while the other ten PCGs used ATG as the initiation codon in *L. boringii*; however, in *L. liui*, by comparison, there was one more PCG (*nad4*) starting with GTG, and the remaining nine PCGs start with ATG. The termination codon usage in *L. boringii* and *L. liui* was diverse: complete codons were found to be used in seven PCGs (TAA for *nad2*, *cox1*, *nad3*, *nad4l*, and *nad5*; TGA for *atp8*; AGG for *nad6*) and incomplete codons by the six other PCGs (TA+ for *nad1* and *atp6*; T++ for *cox2*, *cox3*, *nd4*, and *cytb*) in *L. boringii*, while in *L. liui,* a difference appeared in the usage of complete termination codons (TAA for *nad2*, *cox1*, *nad3*, and *nad4l*; CAT for *atp8*; AGG for *nad5* and *nad6*). Nucleotide composition analysis of the 13 PCGs revealed that they shared similar patterns, except for the *nad6* gene, which had unusual base proportions of T (35.1%), C (12.7%), G (33.7%), and A (18.4%) in *L. boringii* and T (35.5%), C (12.9%), G (32.9%), and A (18.6%) in *L. liui*, respectively; thus, the *nad6* gene presents an extraordinary but positive GC skew (Table 3). There were several distinct but overlapping sites found in the mitogenomes of the two species: in *L. boringii*, these sites ranged from 1 to 28 bp and overlapped at 10 positions, occupying a total length of 50 bp, with the longest overlap (28 bp) located between the *nad5* and *nad6* genes; in *L. liui,* the overlapping sites were found dispersed in nine positions that range from 1 to 7 bp and a total length of 22 bp, with the longest (7 bp) lying between *nad4l* and *nad4*. 

### 3.3. Codon Usage and Genetic Distances

**Figure 3:**
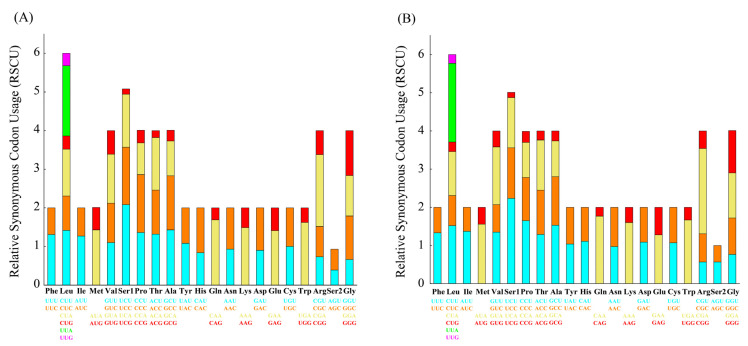
Relative synonymous codon usage (RSCU) of mitogenome of *L. boringii* (**A**) and *L. liui* (**B**).

**Figure 4:**
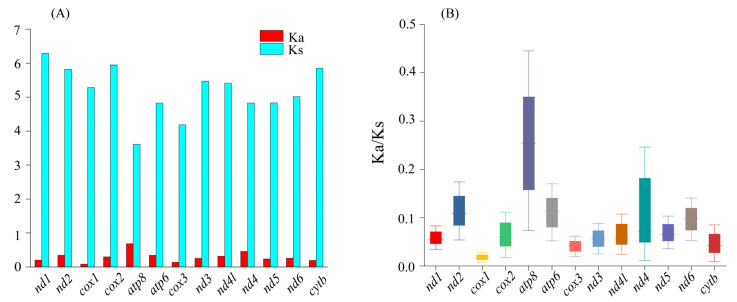
(**A**) The ratio of synonymous (Ks) and non-synonymous (Ka) substitution, and (**B**) Ka/Ks of 13 PCGs among 21 species within Megophryidae.

The codon usage of the PCGs showed an extremely similar pattern that was shared between *L. boringii* and *L. liui* (Figure 3). The UCU (Ser1), CGA (Arg), UUA (Leu), and CAA (Gln) had the highest frequencies in both *L. boringii* and *L. liui*. RSCU analysis of both species indicated that six amino acids (Val, Pro, Thr, Ala, Arg, and Gly) were encoded by four synonymous codons, with the exceptions being Leu and Ser, encoded by six codons, and all others (Phe, Ile, Met, Tyr, His, Gln, Asn, Lys, Asp, Glu, Cys, and Trp) were encoded by two codons (Figure 3). Ka was generally less than Ks, and the average values between species pairs within 21 Megophryidae species ranged from 0 to 0.686 for Ka and from 0 to 6.2922 for Ks (Figure 4A). The Ka/Ks ratios of all the 13 PCGs were less than 1, with the highest Ka/Ks ratio (0.675) in *atp8* (Figure 4B). None of the PCGs showed Ka/Ks ≥ 1, which indicates a generally negative or purifying selection. The *atp8*, *atp6*, and *nad2* genes were found to have relatively fast evolutionary rates, whereas the *cox1*, *cox3*, and *cytb* genes have a relatively slow evolutionary rate. 

**Figure 5:**
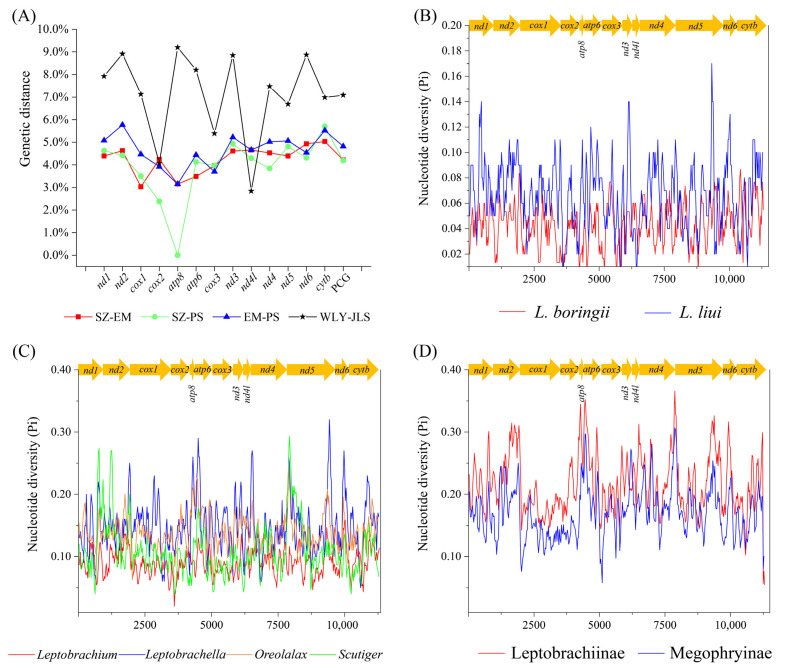
(**A**) Genetic distances between different populations of *L. boringii* and *L. liui*, and nucleotide diversity (Pi) under sliding window analysis of (**B**) three populations of *L. boringii* and two populations of *L. liui*, (**C**) four groups of Clade II, and (**D**) two clades of Megophryidae.

The overall genetic distances of PCGs between two of the three populations of *L. boringii* were 4.82% (EM and PS), 4.22% (EM and SZ), and 4.19% (PS and SZ). While looking at each of the PCGs, *atp8* had the highest genetic variance, followed by *cox2*, *cox1*, and *nad2*, and *cox3* had the lowest genetic variance among the three populations (Figure 5A). As for *L. liui*, the overall genetic distance of PCGs between the two populations (JLS and WLY) was 7.09%, in which *atp8* and *nad4l* had the highest and the lowest genetic variances, respectively (Figure 5A). In addition, when using the sliding window analysis along the concatenated PCG dataset, we found that the nucleotide diversity (Pi) was also highly variable among different gene sequences, ranging from 0.198 (*cox1*) to 0.352 (*atp8*) of 13 PCGs within the 21 species. Generally, the *atp8*, *atp6*, and *nad2* genes had relatively high nucleotide diversity, while the *cox1*, *cox2*, and *cox3* genes had comparatively low nucleotide diversity, no matter the examined sequences, which were grouped from (1) three populations of *L. boringii* and two populations of *L. liui* (Figure 5B), (2) four subgroups of Clade II (Figure 5C), or two major clades of Megophryidae (Figure 5D).

### 3.4. Phylogenetic Analysis

**Figure 6:**
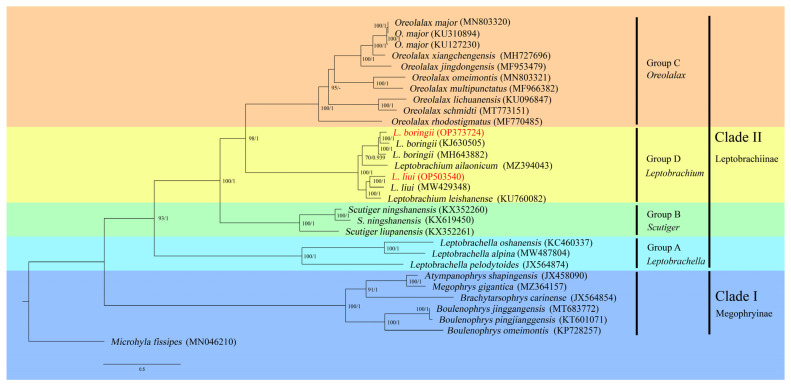
Phylogenetic relationships within Megophryidae derived from ML method based on 13 PCGs. Note: The numbers on the branches represent the bootstrap values and posterior probabilities of ML/BI analyses. The number after species name is the GenBank accession number. Names in red show the phylogenetic positions of *L. boringii* and *L. liui* that we sequenced in this study.

The phylogenetic trees based on both ML and BI methods showed highly consistent topologies, with strong support values in most branches (Figure 6). The genus *Leptobrachium* was divided into two subgroups. *L. boringii* was recovered into the sister group of *L. ailaonicum*, and the three samples of *L. boringii* formed a monophyly, while the PS population branched first and the EM population and SZ population followed (obtained in this study). The two populations (WLY and JLS) of *L. liui* formed a monophylum and were recovered as the sister species of *L. leishanese.* According to the phylogenetic tree, the Megophryidae can be divided into two major clades corresponding to two subfamilies. Clade I (the subfamily Megophryinae) contains species of the following four genera: *Atympanophrys*, *Megophrys, Brachytarsophrys,* and *Boulenophrys.* Clade II (the subfamily Leptobrachiinae) can be further divided into four well-supported major groups in the following order: *Leptobrachella* (Group A) first, followed by *Scutiger* (Group B), then the sister groups *Oreolalax* (Group C) and *Leptobrachium* (Group D) (Figure 6). 

## 4. Discussion

Mitochondrial DNA serves as an efficient molecular marker that has been widely applied in evolution-related studies for a variety of species [40]. Compared with relatively abundant and complex nuclear DNA, the mitochondrial genome has many favorable features, such as simple structure with conserved coding regions, low levels of recombination, multiple copy number, rapid evolutionary rate, and maternal inheritance [40,41]. Therefore, mitochondrial DNA has played a valuable role in reconstructing phylogenetic relationships, revealing population genetic structures, estimating divergence times, identifying relatedness between recently diverged species, etc. [29,42,43,44,45]. 

The traditional method for obtaining a mitogenome is the PCR-targeted method based on chain termination sequencing [46], which has greatly promoted the development of relevant studies. Although the PCR-targeted method is simple to operate, it is time-consuming and primer-dependent, and the mitogenomes combined based on different DNA fragments were usually uncomplete [27,28]. In recent years, the advent of next-generation sequencing (NGS) technology has revolutionized biological studies for producing thousands or even millions of DNA reads within a short period [47,48]. With the ever-increasing throughput and ever-decreasing costs with the development of NGS, more and more complete mitochondrial genomes have been reported and used as essential sources and optimal molecular markers for studies on evolution, phylogenetics, population genetics, and biological conservation [29,43,48,49,50,51]. Although previous studies [27,28] have already reported the mitogenomes of *L. boringii* based on the traditional PCR-targeted method, these mitogenomes were not as complete as what we assembled based on NGS in this study. Nevertheless, the gene organization, base composition pattern, and transcriptional direction were almost the same among the three mitogenomes of *L. boringii*, and they were also similar to those of other congeners [29,52], including *L. liui* that we sequenced and assembled in this study (Table 1, Table 2 and Table 3, Figure 2).

As a result of this study, we report and describe the characteristics of two newly sequenced mitogenomes of *L. boringii* and *L. liui* in more detail. For instance, codon usage bias is a phenomenon in which specific codons are used more frequently than other synonymous codons by certain organisms during the translation of genes to proteins. With rapid progress in whole-genome sequencing, analysis of codon usage bias at the genome level, rather than for a single gene or a set of genes, has been increasing [42]. The results of RSCU analyses indicate that in both *L. boringii* and *L. liui*, codons with A or T at the third position are always overused compared with other synonymous codons (Figure 3). The biased usage of AT nucleotides is also reflected in the form of codon usage, where the highly frequently used codons, UCU (Ser1), CGA (Arg), UUA (Leu), and CAA (Gln), all end with A or T, which might also partly contribute to the higher A+T content. 

The positive selection analyses based on the PCGs of mitogenome can shed light on the question of whether natural selection affected the functional characteristics of the mitochondrion [53]. The ratio of Ka and Ks is a popular proxy to detect adaptive evolution from viruses to humans [54]. It is considered that Ka/Ks > 1 indicates positive selection, Ka/Ks = 1 neutrality, and Ka/Ks < 1 negative or purifying selection [55]. The Ka/Ks value from each of the PCGs within Megophryidae was less than 1 (Figure 4), which indicates that the overall evolution pattern of the mitogenomes of Megophryidae tend to be conservative in maintaining the functions of regularly generated proteins [56]. Interestingly, the Ka/Ks of each of the PCGs indicates that the evolutionary rates are relatively fast for *atp8*, *atp6*, and *nad2* and relatively slow for *cox1*, *cox3,* and *cytb* (Figure 4). This pattern was also revealed in the nucleotide diversity analyses based on different PCGs or from different examined groups under sliding windows (Figure 5). 

The previously known mitogenomes of *L. boringii* and *L. liui* from other populations [27,28,29], and two newly sequenced mitogenomes in this study, together, provided us with an excellent opportunity to examine the intraspecific and interspecific genetic distances among the species of *Leptobrachium*. The intraspecific genetic distances of *L. boringii* were generally greater than 4%, which means the three examined populations are highly differentiated. This trend was also revealed in other studies. For instance, Yang et al. [57] found that *L. boringii* in the Sichuan and Hunan Provinces had significant genetic differentiation with relatively poor gene flow, and the genetic distance between them was 4.7% based on 1097 bp of *cytb* genes. Interestingly, our study further showed that the differentiation pattern of *L. boringii* is not straightforward along the current geographic distances. The PS population (in Chongqing Province) was between the EM (in Sichuan Province) and SZ population (in Hunan Province) from the geographic point of view; however, it divided first and allowed the remaining far-distanced EM and SZ populations to group together in the phylogenetic tree (Figure 6). This suggests the phylogeographic pattern of *L. boringii* among its ranges is shaped by multiple factors. Comparatively, the intraspecific genetic distance between the two populations of *L. liui* was even greater (7.09%) and almost reached that of the interspecific genetic distance between *L. liui* and *L. leishanensis* (7.85%). Although we could identify that the sample we sequenced was a subspecies (*L. liui yaoshanensis*) due to its four keratinized spines [22], we were unable to identify whether or not the JLS population was from another subspecies (*L. liui liui*) because no morphological traits were mentioned in the literature [29] and, additionally, whether the JLS population was in the boundary area of two subspecies. Even so, our results show that the intraspecific genetic distance of *L. liui* is substantially high; thus, further taxonomic studies are of worth. The interspecific genetic distance between *L. boringii* and *L. liui* was relatively high, as they were found in two different subgroups of *Leptobrachium* (Figure 5), which is consistent with another study based on 2 mitochondrial DNA markers (*cytb* and *nad4*) and 11 microsatellite loci [58]. 

While using 27 mitogenome sequences available from NCBI and 2 newly obtained in this study as the ingroups, with *Microhyla fissipes* in the Microhylidae family as the outgroup, a phylogenetic tree within Megophryidae based on mitogenomes was able to be reconstructed in this study (Figure 5). The family Megophryidae was divided into two clades (Clade I and II) corresponding to two subfamilies, Megophryinae and Leptobrachiinae, respectively. Although the species involved here are still limited, the division of two subfamilies is supported. Leptobrachiinae was divided into four groups corresponding to four genera and well supported based on the values: the genus *Leptobrachella* divided first and then *Scutiger*, followed by the sister group of *Leptobrachium* and *Oreolalax*. However, this is not consistent with a previous study based on three mitochondrial and nine nuclear genes, which showed that *Leptobrachella* divided first, followed by *Leptobrachium*, leaving *Scutiger* and *Oreolalax* to form a sister group [5]. This is a reminder that the intergeneric phylogeny within Leptobrachiinae requires further study for verification. When it comes to the phylogenetic relationships within *Leptobrachium*, our study shows that two lineages can be distinguished: one with *L. boringii* and *L. ailaonicum*, and the other one formed by *L. liui* and *L. leishanense*. The relationship of the above two lineages is consistent with the traditional distinction of two species groups according to morphological characteristics, especially the number of keratinized spines (10–48 in *L. boringii* species group vs. 2–6 in *L. liui* species group, [22]). However, this relationship is not well supported by previous studies using relatively limited mitochondrial DNA sequences. For instance, Rao et al. [8] and Matsui et al. [14] revealed a (*L. ailaonicum*, (*L. boringii*, (*L. liui*, *L. leishanense*))) relationship based on 16S rRNA, *nad4*, and *cytb* (a total of 2566 bp), and 12S rRNA, tRNA^Val^, and 16S rRNA (a total of 2009 bp), respectively. Chen et al. [59] recovered a (*L. boringii*, (*L. ailaonicum*, (*L. liui*, *L. leishanense*))) relationship based on 1914 bp of 12S rRNA, tRNA^Val^, and 16S rRNA sequences. Although the phylogenetic relationships would change along with the differences in the DNA markers used, it has been generally recognized that the use of a complete mitogenome is superior to individual genes in uncovering evolutionary relationships [60,61]. However, it has to be noted that the bootstrap value of the node of *L. ailaonicum* and *L. boringii* in our study was still not so supportive (only 70), which could also be treated and collapsed as an unresolved polytomy with a (*L. ailaonicum*, *L. boringii* (*L. liui*, *L. leishanense*)) relationship. In a word, the whole picture of the evolutionary history within the groups of Megophryidae is still as presented until more and more mitogenome data, such as for the two species reported in this study, become available in the future. 

## Figures and Tables

**Table 1:** Characteristics of the mitogenome of *L. boringii* (LB) and *L. liui* (LL).

Gene	Position		Length (bp)		Start Codon		Stop Codon		Anti Codon	Strand *	Intergenic Nucleotide ^#^	
	LB	LL	LB	LL	LB	LL	LB	LL			LB	LL
tRNA^Phe^	1–67	1–67	67	67					GAA	H		
12S rRNA	68–1007	68–1004	940	937						H		
tRNA^Val^	1004–1072	1001–1069	69	69					TAC	H	−4	−4
16S rRNA	1083–2662	1099–2661	1580	1563						H	10	29
tRNA^Leu^	2661–2735	2660–2734	75	75					TAA	H	−2	−2
*nad1*	2736–3709	2735–3708	974	974	ATG	ATG	TA+	TA+		H		
tRNA^Ile^	3712–3782	3711–3781	71	71					CAT	H	2	2
tRNA^Gln^	3782–3852	3781–3851	71	71					TTG	L	−1	−1
tRNA^Met1^	3852–3920	3851–3919	69	69					CAT	H	−1	−1
tRNA^Met2^	4029–4085	4019–4086	57	68					CAT	H	108	99
*nad2*	4096–5139	4087–5130	1044	1044	ATG	ATG	TAA	TAA		H	10	
tRNA^Ala^	5144–5213	5135–5204	70	70					TGC	L	4	4
tRNA^Asn^	5214–5286	5205–5277	73	73					GTT	L		
NCR	5289–5316	5280–5307	28	28						H	2	2
tRNA^Cys^	5316–5379	5307–5370	64	64					GCA	L	−1	−1
tRNA^Tyr^	5380–5449	5371–5440	70	70					GTA	L		
*cox1*	5451–7013	5442–7004	1563	1563	GTG	GTG	TAA	TAA		H	1	1
tRNA^Ser^	7011–7081	7002–7072	71	71					TGA	L	−3	−3
tRNA^Asp^	7086–7153	7077–7144	68	68					GTC	H	4	4
*cox2*	7154–7841	7145–7832	688	688	ATG	ATG	T++	T++		H		
tRNA^Lys^	7842–7915	7833–7906	74	74					TTT	H		
*atp8*	7916–8101	7907–8071	186	165	GTG	GTG	TAA	CAT		H		
*atp6*	8092–8774	8083–8765	683	683	ATG	ATG	TA+	TA+		H	−10	11
*cox3*	8774–9557	8765–9548	784	784	ATG	ATG	T++	T++		H	−1	−1
tRNA^Gly^	9558–9626	9549–9617	69	69					TCC	H		
*nad3*	9627–9971	9618–9962	345	345	GTG	GTG	TAA	TAA		H		
tRNA^Arg^	9970–10,038	9961–10,029	69	69					TCG	H	−2	−2
*nad4l*	10,039–10,335	10,030–10,326	297	297	ATG	ATG	TAA	TAA		H		
*nad4*	10,329–11,706	10,320–11,697	1378	1378	ATG	GTG	T++	T++		H	−7	−7
tRNA^His^	11,707–11,775	11,698–11,766	69	69					GTG	H		
tRNA^Ser^	11,776–11,842	11,767–11,833	67	67					GCT	H		
tRNA^Leu^	11,843–11,915	11,834–11,906	73	73					TAG	H		
*nad5*	11,947–13,779	11,908–13,737	1833	1830	ATG	ATG	TAA	AGG		H	31	1
*nad6*	13,752–14,261	13,743–14,252	510	510	ATG	ATG	AGG	AGG		L	−28	5
tRNA^Glu^	14,262–14,330	14,253–14,321	69	69					TTC	L		
*cytb*	14,335–15,475	14,326–15,466	1141	1141	ATG	ATG	T++	T++		H	4	4
tRNA^Thr^	15,476–15,545	15,467–15,536	70	70					TGT	H		
tRNA^Pro^	15,548–15,616	15,539–15,607	69	69					TGG	L	2	2
tRNA^Trp^	15,626–15,694	15,618–15,686	69	69					TCA	H	9	10
D-loop	15,695–17,100	15,687–17,501	1406	1815						H	0	0

* H and L indicate genes transcribed on the heavy and light strand, respectively. ^#^ Positive numbers correspond to the nucleotides separating adjacent genes; negative numbers indicate overlapping nucleotides.

**Table 2:** Base composition (in percentages) of the mitogenomes of three populations of *L. boringii*, two populations of *L. liui,* and another 21 species in Megophryidae.

Species	Total Length (bp)	T%	C%	G%	A%	A+T%	AT Skew	GC Skew	Accession Number
*L. boringii* (SZ)	17,100	31.5	25.5	15.3	27.7	59.2	−0.064	−0.250	OP373724 *
*L. boringii* (EM)	17,085	31.5	25.5	15.4	27.6	59.1	−0.066	−0.247	KJ630505
*L. boringii* (PS)	16,557	31.6	25.5	15.2	27.7	59.3	−0.066	−0.253	MH643882
*L. liui* (WLY)	17,501	32.7	24.3	14.8	28.1	60.8	−0.076	−0.243	OP503540 *
*L. liui* (JLS)	17,190	32.6	24.4	14.9	28.1	60.7	−0.074	−0.242	MW429348
*Oreolalax major*	17,786	32.6	24.3	14.3	28.7	61.3	−0.064	−0.259	MN803320
*O. major*	15,469	32.1	24.7	14.4	28.7	60.8	−0.056	−0.263	KU310894
*O. major*	17,431	32.4	24.5	14.4	28.8	61.2	−0.059	−0.260	KU127230
*Oreolalax xiangchengensis*	17,110	33.0	23.6	14.2	29.2	62.2	−0.061	−0.249	MH727696
*Oreolalax jingdongensis*	17,864	32.7	23.9	14.3	29.1	61.8	−0.058	−0.251	MF953479
*Oreolalax omeimontis*	17,675	32.6	25.0	14.0	28.5	61.1	−0.067	−0.282	MN803321
*Oreolalax multipunctatus*	17,358	32.0	24.2	14.3	28.5	60.5	−0.058	−0.257	MF966382
*Oreolalax lichuanensis*	17,702	32.2	24.9	15.0	28.0	60.2	−0.070	−0.248	KU096847
*Oreolalax schmidti*	18,481	32.8	24.5	14.4	28.3	61.1	−0.074	−0.260	MT773151
*Oreolalax rhodostigmatus*	18,676	32.4	24.9	14.7	28.0	60.4	−0.073	−0.258	MF770485
*Leptobrachium ailaonicum*	17,318	31.8	25.0	15.3	27.9	59.7	−0.065	−0.241	MZ394043
*Leptobrachium leishanense*	17,485	32.6	24.4	14.9	28.1	60.7	−0.074	−0.242	KU760082
*Scutiger ningshanensis*	17,265	32.8	24.2	14.0	29.1	61.9	−0.060	−0.267	KX619450
*S. ningshanensis*	16,799	32.6	24.3	14.3	28.8	61.4	−0.062	−0.259	KX352260
*Scutiger liupanensis*	16,890	32.2	24.9	14.5	28.4	60.6	−0.063	−0.264	KX352261
*Leptobrachella oshanensis*	17,747	29.9	26.3	15.2	28.8	58.7	−0.019	−0.267	KC460337
*Leptobrachella alpina*	17,763	30.8	25.6	15.1	28.5	59.3	−0.039	−0.258	MW487804
*Leptobrachella pelodytoides*	14,682	29.1	27.8	15.5	27.7	56.8	−0.025	−0.284	JX564874
*Atympanophrys shapingensis*	17,631	31.5	26.0	14.3	28.2	59.7	−0.055	−0.290	JX458090
*Megophrys gigantica*	18,259	32.1	25.2	14.3	28.4	60.5	−0.061	−0.276	MZ364157
*Brachytarsophrys carinense*	15,271	29.8	27.6	15.1	27.5	57.3	−0.040	−0.293	JX564854
*Boulenophrys jingganggensis*	17,263	31.6	26.3	14.5	27.6	59.2	−0.068	−0.289	MT683772
*Boulenophrys pingjianggensis*	17,866	32.0	25.9	14.3	27.8	59.8	−0.070	−0.289	KT601071
*Boulenophrys omeimontis*	17,013	31.8	25.7	14.2	28.3	60.1	−0.058	−0.288	KP728257

* the sequence obtained in this study.

**Table 3:** Nucleotide composition and skewness of different regions in the mitogenomes of *L. boringii* (LB) and *L. liui* (LL).

	Length (bp)		T%		C%		G%		A%		A+T%		AT Skew		GC Skew	
	LB	LL	LB	LL	LB	LL	LB	LL	LB	LL	LB	LL	LB	LL	LB	LL
*nad1*	974	974	34.7	35.3	24.8	23.8	14.8	14.5	25.7	26.4	60.4	61.7	−0.149	−0.145	−0.253	−0.244
*nad2*	1044	1044	32.3	32.6	29.7	29.4	12.1	12.2	26.0	25.9	58.3	58.4	−0.108	−0.115	−0.421	−0.415
*cox1*	1563	1563	31.6	33.1	24.7	22.5	18.0	17.8	25.7	26.6	57.3	59.7	−0.103	−0.110	−0.157	−0.117
*cox2*	688	688	31.4	32.6	25.4	24.3	15.7	15.4	27.5	27.8	58.9	60.3	−0.065	−0.080	−0.236	−0.223
*atp8*	186	165	38.7	43.6	24.2	20.6	10.2	9.7	26.9	26.1	65.6	69.7	−0.180	−0.252	−0.407	−0.360
*atp6*	683	683	35.7	36.0	26.2	25.3	13.6	12.0	24.5	26.6	60.2	62.7	−0.186	−0.150	−0.317	−0.357
*cox3*	784	784	33.9	33.8	25.1	25.8	16.7	16.8	24.2	23.6	58.1	57.4	−0.167	−0.178	−0.201	−0.210
*nad3*	345	345	38.3	39.1	25.2	23.8	15.4	14.2	21.2	22.9	59.5	62.0	−0.287	−0.262	−0.241	−0.252
*nad4l*	297	297	35.4	37.4	27.9	25.9	12.8	12.8	23.9	23.9	59.3	61.3	−0.194	−0.220	−0.371	−0.339
*nad4*	1378	1378	33.4	35.2	28.4	26.1	12.7	12.3	25.5	26.4	58.9	61.6	−0.134	−0.143	−0.382	−0.357
*nad5*	1833	1830	33.8	34.8	26.0	25.0	13.1	12.9	27.0	27.4	60.8	62.1	−0.112	−0.119	−0.330	−0.319
*nad6*	510	510	35.1	35.5	12.7	12.9	33.7	32.9	18.4	18.6	53.5	54.1	−0.312	−0.312	0.453	0.436
*cytb*	1141	1141	34.1	35.1	27.0	26.1	14.7	13.7	24.2	25.1	58.3	60.2	−0.170	−0.167	−0.295	−0.313
PCGs	11,426	11,402	33.7	34.7	25.8	24.6	15.3	14.9	25.2	25.8	58.9	60.5	−0.144	−0.148	−0.255	−0.246
PCGs^–1st^	3809	3801	33.9	35.0	27.0	26.0	14.9	14.3	24.2	24.6	58.1	59.6	−0.167	−0.174	−0.289	−0.290
PCGs^–2nd^	3809	3801	34.7	35.4	26.0	24.9	14.2	14.3	25.2	25.4	59.9	60.8	−0.159	−0.164	−0.294	−0.270
PCGs^–3rd^	3808	3800	32.6	33.7	24.3	22.9	16.8	16.1	26.2	27.3	58.8	61.0	−0.109	−0.105	−0.182	−0.174
12S rRNA	940	937	25.0	25.5	23.7	23.5	19.9	19.9	31.4	31.2	56.4	56.7	0.113	0.100	−0.087	−0.084
16S rRNA	1580	1563	27.0	27.2	21.8	21.8	17.5	17.1	33.7	34.0	60.7	61.2	0.110	0.111	−0.109	−0.120
rRNAs	2520	2500	26.3	26.6	22.5	22.4	18.2	18.1	33.0	32.9	59.3	59.5	0.113	0.107	−0.106	−0.106
tRNAs	1536	1536	28.6	28.6	20.4	20.8	22.1	21.7	28.8	29.0	57.4	57.6	0.003	0.007	0.040	0.021
D-loop	1406	1815	35.1	38.8	21.2	19.4	13.8	12.8	29.9	29.0	65.0	67.8	−0.080	−0.145	−0.211	−0.205
Mitogenome	17,100	17,501	31.5	32.7	25.5	24.3	15.3	14.8	27.7	28.1	59.2	60.8	−0.064	−0.076	−0.250	−0.242

## Data Availability

The assembled mitogenome sequences were deposited in NCBI (https://www.ncbi.nlm.nih.gov/ (accessed on 1 October 2022)) with accession numbers: OP373724 and OP503540. All data generated by this study are available from the corresponding author upon reasonable request.
